# A patient on RIPE therapy presenting with recurrent isoniazid-associated pleural effusions: a case report

**DOI:** 10.1186/1752-1947-5-558

**Published:** 2011-11-30

**Authors:** Vanja Varenika, Paul D Blanc

**Affiliations:** 1 Department of Occupational and Environmental Medicine, University of California, San Francisco, 350 Parnassus Avenue, Room 609, San Francisco, CA 94143-0924, USA

## Abstract

**Introduction:**

The clinical scenario of a new or worsening pleural effusion following the initiation of antituberculous therapy has been classically referred to as a 'paradoxical' pleural response, presumably explained by an immunological rebound phenomenon. Emerging evidence suggests that there also may be a role for a lupus-related reaction in the pathophysiology of this disorder.

**Case presentation:**

An 84-year-old Asian man treated with isoniazid, along with rifampin, pyrazinamide and ethambutol for suspected extrapulmonary tuberculosis, presented with a recurrent pleural effusion, his third episode since the initiation of this therapy. The first effusion occurred one month after the start of treatment, without any prior evidence of pulmonary tuberculosis involvement. Follow-up testing, including thoracoscopic pleural biopsies, never confirmed tuberculosis infection. Further evaluation yielded serological evidence suggesting drug-induced lupus. No effusions recurred following the discontinuation of isoniazid, although other antituberculosis medications were continued.

**Conclusion:**

The immunological rebound construct is inconsistent with the evolution of this case, which indicates rather that drug-induced lupus may explain at least some cases of new pleural effusions following the initiation of isoniazid.

## Introduction

Pleural effusions are frequently observed in pulmonary *Mycobacterium tuberculosis *infection (TB), occurring in up to 30% of cases [1]. New onset pleural effusions following weeks of antituberculous treatment are far less common. This clinical scenario, of a delayed emerging effusion following the initiation of therapy, has been referred to as a 'paradoxical' response [2,3]. Published reports often attribute this to an immunological rebound, in which an excessive antigen load from bacteriolysis is presumed to interact with a 'revved-up' cell-mediated immunity. This model of a pleural-based response draws on immunological phenomena related to tuberculous lymphadenitis and intracranial tuberculoma [4]. While this has been the traditional explanation, cases have emerged that suggest a role for drug-induced lupus mediating this phenomenon in at least some presentations of delayed effusions following the initiation of TB treatment. This report highlights a case of recurrent pleural effusions stemming from isoniazid-induced lupus and discusses the inherent problems with the presumption that the paradoxical pleural response to anti-TB therapy explains all effusions arising during such treatment.

## Case presentation

An 84-year-old Asian man was admitted to hospital for dyspnea and was found to have a large, recurrent right-sided pleural effusion. This was the third time our patient presented with an effusion since the initiation of rifampin, isoniazid, pyrazinamide and ethambutol (RIPE) therapy for presumed extrapulmonary TB. Nine months prior to this presentation, our patient underwent a screening colonoscopy with a biopsy that reportedly yielded a single granulomatous, acid-fast stain-positive, but TB culture-negative lesion. No anti-TB treatment was initiated at that time. The purportedly acid-fast stain-positive specimen could not be located subsequently for re-review. A repeat colonoscopy with biopsy at six months demonstrated no granulomas or other evidence of TB. There was no radiographic evidence of pulmonary TB and interval sputum cultures were negative, as was a serum QuantiFERON-TB Gold test. Nevertheless, our patient was started on RIPE combination therapy.

**Figure 1:**
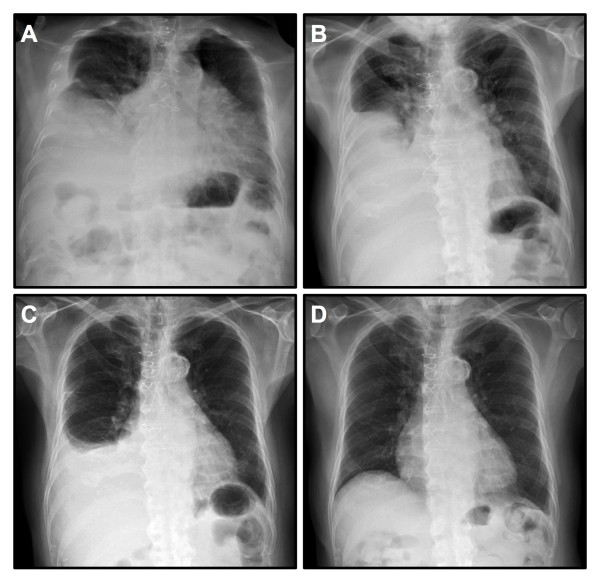
**Chest X-ray images**. **(A) **Large pleural effusion in his right lung field found on the first admission. **(B) **Large pleural effusion in his right lung field on the second admission. **(C) **Pleural effusion in his right lung field on the current admission, two weeks after the last previous thoracocentesis. **(D) **Clear chest X-ray six months after the discontinuation of isoniazid.

Four weeks after the initiation of this regimen (two months prior to the episode described in this report), our patient complained of dyspnea, cough and weakness. He was admitted to hospital with the initial right-sided pleural effusion presentation (Figure 1). Thoracocentesis yielded exudative, culture- and stain-negative pleural fluid (Table 1). Our patient was given a presumptive diagnosis of a paradoxical tuberculous effusion and discharged on a tapering course of systemic glucocorticosteroids while he continued on RIPE therapy. Two months later, he was re-admitted with a similar clinical picture. A further liter of exudative fluid was removed by repeat thoracocentesis (Figure 1 and Table 1).

**Table 1:** Laboratory values for the pleural fluid collected from the three effusions our patient manifested.

	1st Effusion	2nd Effusion	3rd Effusion
**Color**	Red	Red	Yellow
**Red blood cells (×10^9^/L)**	57.25	107	5.575
**White blood cells (×10^9^/L)**	2.075	0.219	0.17
**Neutrophils**	11%	1%	1%
**Lymphocytes**	78%	41%	96%
**Monocytes**	11%	55%	2%
**Eosinophils**	0%	3%	1%
**pH**	7.81	-	-
**Albumin (g/dL)**	2.1	-	-
**Amylase (U/L)**	-	-	67
**Glucose (mg/dL)**	257	212	125
**Protein (g/dL)**	4.2	3.3	3.3
**Lactate dehydrogenase (IU/L)**	104	200	224
**Triglyceride (mg/dL)**	-	-	21
**Complement (CH50)**	-	-	10
**Adenosine deaminase (U/L)**	-	-	23.8
**ANA Titer**	-	-	<1:40
**Serum lactate dehydrogenase (IU/L)**	159	-	-
**Serum Protein (g/dL)**	6.2	-	-

Note: Dash (-) indicates that the test was not performed on the sample in question.

Two weeks later, a re-accumulated pleural effusion led to the admission we report (Figure 1). Our patient underwent a video-assisted thoracoscopic surgical biopsy. The pleural fluid again demonstrated a lymphocytosis with benign cytology and an adenosine deaminase assay yielded a value of 23.8 units per liter (Table 1). Multiple pleural biopsies demonstrated inflammation without any well-formed granulomata. All cultures of pleural fluid and biopsies were TB negative. Our patient's pleural fluid, which had not been tested serologically on previous thorocenteses, manifested a low complement with a total complement activity (CH50) of 10, but was antinuclear antibody (ANA) negative. The serum ANA titer, however, was elevated to 1:160 with a speckled pattern, while a serum antihistone assay was negative. The isoniazid was discontinued, although rifampin and ethambutol were continued to complete a nine-month course as recommended by the infectious disease subspecialty consultants. There have been no further pleural effusions over two years of follow-up.

## Discussion

The application of immunological rebound as an explanation for this case, in which the anti-TB therapy was empiric and without a convincingly demonstrated infection, is not consistent with the specific clinical data in hand. Moreover, the adenosine deaminase value we observed was below the cut-off point (<35U/L) associated with a greater than 90% negative predictive value for pleural TB, even if the pre-test likelihood of disease was as high as 70% [5]. A close examination of similar cases indicates that new effusions can follow the initiation of isoniazid therapy and resolve once that drug is discontinued [2,3]. Given the well-established association of isoniazid with drug-induced lupus (DIL), a lupus-related explanation of therapy-associated onset of pleural effusion in TB is quite plausible and avoids the presumed paradox of immunological rebound. Of note, serositis is a common feature of DIL, with pleural effusions occurring in as many as 50% of cases, depending on the inciting drug. DIL serologies were documented in three previous cases of pleural effusions after anti-TB therapy involving isoniazid [6,7]. These cases showed a markedly decreased CH50 in pleural fluid and an elevated ANA in both the serum and the pleural fluid. Pericardial effusions resulting from isoniazid-induced lupus have also been reported [8]. The non-specific ANA serum elevation we observed is consistent with DIL, as was the pleural effusion resolution following the removal of isoniazid from the treatment regimen. Although we did not observe positive serum antihistone antibodies, these can be negative in as many as a quarter of DIL cases [9]. While DIL is occasionally associated with rifampin, the resolution of symptoms in the case despite continued exposure to this agent argues that it did not play a role [10].

## Conclusion

The case we describe, in conjunction with other cases in the medical literature, underscores the likely pivotal role for DIL in at least some new or worsening effusions during isoniazid treatment. Isoniazid-induced lupus should be considered in the differential diagnosis of a treatment emergent effusion and appropriate diagnostic tests should be pursued before such an effusion is attributed to a paradoxical pleural response.

## Abbreviations

ANA: anti-nuclear antibody; DIL: drug-induced lupus; RIPE: rifampin, isoniazid, pyrazinamide, ethambutol; TB: *Mycobacterium tuberculosis *infection.

## Consent

Written informed consent was obtained from the patient for publication of this case report and any accompanying images. A copy of the written consent is available for review by the Editor-in-Chief of this journal.

## Competing interests

The authors declare that they have no competing interests.

## Authors' contributions

Both VV and PB were directly involved in the care of the patient during the admission described in this report. VV was a major contributor in writing the manuscript with significant overall guidance and editorial assistance from PB. Both authors read and approved the final manuscript.

## References

[B1] Ferrer J Pleural tuberculosis Eur Respir J 1997 10 942 947 9150338

[B2] Singh SK Ahmad Z Pandey DK Gupta V Naaz S Isoniazid causing pleural effusion Indian J Pharmacol 2008 40 2 87 88 10.4103/0253-7613.41045 21279173 PMC3025133

[B3] Gupta RC Dixit R Purohit SD Saxena A Development of pleural effusion in patients during anti-tuberculous chemotherapy: analysis of twenty-nine cases with review of literature Indian J Chest Dis Allied Sci 2000 42 161 166 11089320

[B4] Al-Majed SA Study of paradoxical response to chemotherapy in tuberculous pleural effusion Respir Med 1996 90 211 214 10.1016/S0954-6111(96)90289-9 8736654

[B5] Porcel JM Esquerda A Bielsa S Diagnostic performance of adenosine deaminase activity in pleural fluid: a single-center experience with over 2100 consecutive patients Eur J Int Med 2010 21 419 423 10.1016/j.ejim.2010.03.011 20816597

[B6] Hiraoka K Nagata N Kawajiri T Suzuki K Kurokawa S Kido M Sakamoto N Paradoxical pleural response to antituberculous chemotherapy and isoniazid-induced lupus. Review and report of two cases Respiration 1998 65 152 155 10.1159/000029251 9580929

[B7] Jouneau S Volatron AC Brinchault G Morel V Belleguic C Delaval P Pleuro-péricardite induite par l'isoniazide [Isoniazid-induced pleuro-pericarditis] Rev Pneumol Clin 2003 59 6 357 359 14745341

[B8] Siddiqui MA Khan IA Isoniazid-induced lupus erythematosus presenting with cardiac tamponade Am J Therapurt 2002 9 163 165 10.1097/00045391-200203000-00012 11897931

[B9] Katz U Zandman-Goddard G Drug-induced lupus: an update Autoimmun Rev 2010 10 46 50 10.1016/j.autrev.2010.07.005 20656071

[B10] Patel DG Anstey AV Rifampin-induced lupus erythematosus Clin Expir Derm 2001 26 60 62 10.1046/j.1365-2230.2001.00809.x 11422169

